# Transcriptomic Analysis of Metabolic Pathways in Milkfish That Respond to Salinity and Temperature Changes

**DOI:** 10.1371/journal.pone.0134959

**Published:** 2015-08-11

**Authors:** Yau-Chung Hu, Chao-Kai Kang, Cheng-Hao Tang, Tsung-Han Lee

**Affiliations:** 1 Ph.D. Program in Microbial Genomics, National Chung Hsing University, Taichung, and Academia Sinica, Taipei, Taiwan; 2 Tainan Hydraulics Laboratory, National Cheng Kung University, Tainan, Taiwan; 3 Graduate Institute of Marine Biology, National Dong Hwa University, Pingtung, Taiwan; 4 National Museum of Marine Biology and Aquarium, Pingtung, Taiwan; 5 Department of Life Sciences, National Chung Hsing University, Taichung, Taiwan; 6 Department of Biological Science and Technology, China Medical University, Taichung, Taiwan; National Cheng Kung University, TAIWAN

## Abstract

Milkfish (*Chanos chanos*), an important marine aquaculture species in southern Taiwan, show considerable euryhalinity but have low tolerance to sudden drops in water temperatures in winter. Here, we used high throughput next-generation sequencing (NGS) to identify molecular and biological processes involved in the responses to environmental changes. Preliminary tests revealed that seawater (SW)-acclimated milkfish tolerated lower temperatures than the fresh water (FW)-acclimated group. Although FW- and SW-acclimated milkfish have different levels of tolerance for hypothermal stress, to date, the molecular physiological basis of this difference has not been elucidated. Here, we performed a next-generation sequence analysis of mRNAs from four groups of milkfish. We obtained 29669 unigenes with an average length of approximately 1936 base pairs. Gene ontology (GO) analysis was performed after gene annotation. A large number of genes for molecular regulation were identified through a transcriptomic comparison in a KEGG analysis. Basal metabolic pathways involved in hypothermal tolerance, such as glycolysis, fatty acid metabolism, amino acid catabolism and oxidative phosphorylation, were analyzed using PathVisio and Cytoscape software. Our results indicate that in response to hypothermal stress, genes for oxidative phosphorylation, e.g., succinate dehydrogenase, were more highly up-regulated in SW than FW fish. Moreover, SW and FW milkfish used different strategies when exposed to hypothermal stress: SW milkfish up-regulated oxidative phosphorylation and catabolism genes to produce more energy budget, whereas FW milkfish down-regulated genes related to basal metabolism to reduce energy loss.

## Introduction

The development of next-generation sequencing (NGS), high throughput sequencing analysis, and associated bioinformatic analyses has enabled considerable advances in our understanding of a range of biochemical and physiological processes. NGS has been used to investigate environmental adaptation in model and non-model fish species, e.g., the muscle transcriptome of gilthead sea bream (*Sparus aurata*) under low temperatures [1], the responses to changes in environmental calcium ion levels in gills of the green spotted pufferfish (*Tetraodon nigroviridis*) [2], the effects of temperature and low oxygen stresses on the liver of the Atlantic salmon (*Salmo salar*) [3], the effects of high temperature on various organs of the Antarctic cryopelagic notothenioid fish (*Pagothenia borchgrevinki*) [4], the muscle transcriptome in the zebrafish (*Danio rerio*) at different temperatures [5], and the effects of exposure to cold in zebrafish larvae [6]. *De novo* assembled transcriptomes reffect the sets of genes that are functionally active under particular conditions. Sequencing of a range of transcriptomes from any given species enables the construction of a genome-wide gene expression profile; it also allows quantification of transcript abundance in order to compare molecular differences that might underlie the mechanisms of environmental adaptation [7, 8].

Changes in ambient temperatures occur both daily and seasonally, and have considerable effects on fish physiology as well as their behavior. Because fish are ectothermic animals, it is important that their biochemical and metabolic processes can adapt to changes in environmental temperatures. Regulation of these processes requires metabolic energy for maintaining homeostasis; thus, a sufficient and reliable energy supply through aerobic or anaerobic metabolic pathways is essential. In fish, various basic metabolic pathways are involved in energy production and are vital to successful compensation and acclimation under stressful temperature conditions. In the gilthead sea bream, the impact of exposure to low seawater (SW) temperature was examined by determining the maximum activities of enzymes involved in glycolysis, the citric acid cycle and lipid oxidation; a microarray analysis indicated that changes in these metabolic pathways played an essential role in coping with cold shock stress in this SW teleost [9].

In a similar manner as temperature response, salinity adaptation also requires physiological compensation and acclimation. During smoltification, salmon adjust their physiological status in order to adapt to SW. The metabolism of the liver, gill, and kidney tissues of smolts is significantly different to that of parr in fresh water (FW) [10–12]. Seear et al. [13] also reported the up-regulation of metabolic genes in the gill tissue of the smolts.

The Na^+^, K^+^-ATPase (NKA) enzyme, which pumps sodium out of the cell and potassium in, accounts for about one-fifth of the total cell energy consumption and plays a major role as the driving force for osmoregulation [14]. Water temperature can affect gill NKA activity in fish, with a general trend towards reduced enzyme activity at lower temperatures [15, 16]. Whether the effects of salinity and temperature on fish are interactive is still uncertain. Low temperature causes shifts in plasma ionic concentrations to those of the environment in blackchin tilapia, *Sarotherodon melanotheron* [17], and alewife, *Alosa pseudoharengus* [18], but no interactions have been reported. However, blue tilapias, *Oreochromis aureus*, can survive in isosmotic media (11.6‰) at lower temperatures than in other salinities, i.e., an isosmotic medium decreases salinity stress at the lower temperatures [19]. Interaction of salinity and temperature on metabolic responses has also been analyzed in the gilthead sea bream; it was found that extremely low temperature affects the energy supply for osmoregulation in this species [20]. Thus, environmental salinities may be a decisive factor affecting energy metabolism for fish survival under hypothermia.

Milkfish (*Chanos chanos*), a common species in the subtropical zone, is a SW euryhaline but is also noted as being stenothermic [21]. Milkfish can compensate for salinity challenges by modulation of their metabolic rates [22] and NKA activities [23]; such modulations enable milkfish to be extremely euryhaline. Milkfish are an important marine aquaculture species in Taiwan and have been cultured for over 300 years. However, fish in cultures in southern Taiwan often die in large numbers during the winter during cold snaps. Hsieh and Kuo [24, 25] measured some aspects of metabolic enzyme activity in milkfish exposed to low temperature and compared these to the carp, a eurythermal FW species. Milkfish NKA activity is lower in SW than in FW at 28°C and 18°C [26; 23]. Although NKA activity in cells from fish in FW at 18°C group could not be restored by *in vitro* incubation at a higher temperature, the higher activity of NKA in FW milkfish indicates the requirement for more energy. The contribution of energy consumptions to basal metabolism are closely correlated to survival. It is well understood that ectoderms modify their metabolic responses to sustain the locomotion, neurotransmission, osmoregulation, and reproduction [27–30]. Salinities affected the hypothermal tolerating ability of the milkfish. The critical thermal minimum for SW milkfish is 13°C compared to 15°C in FW milkfish [26]. Thus, gill, brain, liver, and kidney which are full of glycogen accumulation and highly metabolic organs [30–34] were sampled in this study. The tolerance of milkfish to hypothermal stress is yet to be clarified. For this reason, we compared the transcriptomes of SW- and FW-acclimated milkfish using NGS and investigated the differences in their strategies for energy compensation under hypothermal stress.

## Materials and Methods

### Ethics statement

The experimental protocol was reviewed and approved by the Institutional Animal Care and Use Committee of the National Chung Hsing University (IACUC approval no. 98–110 to THL).

### Fish and experimental treatments

Milkfish were purchased from a local farm in Lukang, Changhua county, Taiwan. Milkfish weighing about 10 g were purchased from a local culture farm and maintained in 500 L closed circuit aquaria in FW and artificial SW (35‰) prepared from FW with the appropriate amounts of RealOcean Synthetic Sea Salt (Camarillo, CA, USA); the fish were kept at 28 ± 1°C and a 12h:12h photoperiod for a month before commencement of the experiments. The fish were fed a daily diet of commercial pellets. For the transcriptomic analyses, 8 fish of each group were transferred to a 100-L tank. Two groups (i.e., SW and FW) of milkfish were treated with low temperatures (hypothermal group). The culture temperature was decreased by 2°C per hour until it had reached 18°C; the two groups of fish were kept at this temperature for 1 week. Two groups of fish (SW and FW) were assigned as control groups and maintained at 28°C.

### Total RNA extraction for NGS

The brain, gills, liver, and kidneys of each fish were collected, frozen in liquid nitrogen, and stored at -80°C until RNA extraction. RNA extraction was carried out using a TriPure Isolation Reagent kit (Roche, Basel, Switzerland) according to the manufacturer’s instructions. After DNase treatment and purification through a spin column (illustra, GE Healthcare, Piscataway, NJ, USA), the RNA was dissolved in RNase-free water. After checking RNA quantity and quality by NanoDrop 2000 spectrophotometer (Thermo, Wilmington, DE, USA), RNA samples from brains, gills, livers and kidneys were pooled in each group. The RNA samples of brains, gills, livers and kidneys in each group were pooled. The pooled RNA samples were mixed to produce four library groups: 28°C/SW, 28°C/FW, 18°C/SW, and 18°C/FW. The four samples were checked again for their integrity and size distribution of 18/28S rRNA by NanoDrop 2000 spectrophotometer (Thermo), agarose gel electrophoresis and Experion automated electrophoresis system. Purified RNA with an A_260_/A_280_ ratio between 1.8 and 2.0 was used. They were then subjected to next-generation sequencing (NGS) by Yourgene Corporation (Taipei, Taiwan). The *de novo* assembly for the whole milkfish transcriptome is derived from these four library groups. Differential expression of the transcriptomes was analyzed by comparing 28°C/FW with 18°C/FW, and 28°C/SW with 18°C/SW.

### Library construction

Poly-A containing mRNA molecules were purified using poly-T oligo-attached magnetic beads for eukaryotic mRNA. A fragmentation mix was added to break the mRNA sequences into small segments. First-strand cDNA was synthesized using these mRNA fragments as RT templates. Second-strand cDNA was synthesized using dNTPs (dUTP replaced dTTP), buffer, RNaseH, and DNA polymerase I. The cDNA templates were purified using a Qiagen kit (Venlo, Netherlands) followed by end repair, poly A tailing, and adaptor connection. The samples then were treated with the USER (Uracil-Specific Excision Reagent) Enzyme (NEB, Massachusetts, U.S.A) to digest the antisense strand DNA as insert size of 200 to 300 bases, and then used for PCR amplification [35]. Finally, the library was sequenced using an IlluminaHiSeq 2000. After sequencing, reads 1 from the sense strand and reads 2 from the anti-sense strand were obtained. The first step in the trim process was to convert the quality score (Q) to error probability. Next, for every base a new value was calculated using the formula: 0.01—Error probability

### *De novo* assembly

The quality trimmed clean reads after removal of control sequences were used for transcriptome assembly of each sample by Trinity with the following setting: maximum distance between read pairs of 500 [36]. Trinity, developed at the Broad Institute and the Hebrew University of Jerusalem, is a novel method for the efficient and robust *de novo* reconstruction of transcriptomes from NGS data. It combines three independent software modules: Inchworm, Chrysalis, and Butterfly, applied sequentially to process large volumes of NGS reads. Trinity partitions the sequence data into many individual de Bruijn graphs, each representing the transcriptional complexity at a given gene or locus, and then processes each graph independently to extract full-length splicing isoforms and to separate transcripts derived from paralogous genes called “contig sequences”.

After assembly of each sample was finished, we used Oases to assemble the transcriptome sequences of the four samples with setting of k-mer 55 and strand specific [37]. All contig sequences generated by Oases were in FASTA format (transcripts.fa) and the description line of the FASTA format. The contig sequence with the highest confidence score at the same “Locus” (genetic region) was picked as the unigene (Unigene.fa). If the contig sequences at the same “Locus” had the same confidence scores, we picked the longest one as the “unigene”. *De novo* assembly was further conducted using the transcriptome assembly tools with parameters as followed: automatic word and bubble size, minimal contig length = 200, perform scaffolding and auto-detected paired distance in CLC Genomics Workbench platform (CLCbio, Anrhus N, Denmark) to generate multiple transcript sets. The unigene (Unigene.fa) was imported and assembled with default settings. Global alignment was performed to refine similar sequence numbers and construct conservative sequences as “transcript”.

The performance of these tools was assessed according to N50 value, mean length, maximum length, and transcript/scaffold numbers. Merging transcripts from different assemblies can also be performed using Oases. We mapped the trimmed reads of the sample to the unigene sequences (transcript sequences) by Bowtie 2 and using a gapped alignment mode [38]. This method was essential for variant discovery because the sequence reads may contain insertion-deletion polymorphisms (INDELs). Without this alignment approach, a read might still be mapped onto the correct position but with consecutive mismatches at INDEL locations. After alignment, we quantified gene expression by eXpress and placed the results in an EXCEL file (Gene_expression.xlsx) [39]. The special term in the EXCEL file is FPKM, which is defined as “expected fragments per kilobase of transcript per million fragments sequenced” [40].

### Gene annotation, GO and KEGG analysis, and metabolic pathway speculations

The assembled unigenes were used to search the NCBI nr protein database (http://www.ncbi.nlm.nih.gov, release date April 15, 2012) using blastx and blastn tools, respectively. E-values, 1^E-5^, indicating sequence conservation and the best alignments were used to annotate the unigenes. The outputs of the blast searches of the NCBI nr protein database were imported into the Blast2GO program for GO term mapping. The results of the Blast2GO analysis were submitted to WEGO for GO classification of biological process, molecular function, and cellular component ontologies. KEGG annotation was performed using the single-directional best-hit (SBH) method in the KAAS web server. This tool is able to assign KEGG Orthology (KO) identifiers or K numbers to query sequences according to sequence similarity and to perform pathway mapping and BRITE mapping processes. The KO system is structured as a four level hierarchy. The top level consists of the following six categories: metabolism, genetic information processing, environmental information processing, cellular processes, organismal systems, and human diseases. Each top level category contains a wide array of sub-categories (the second level). The third level corresponds directly to the KEGG pathways, and the fourth level consists of the leaf nodes representing the functional terms. Basal metabolic pathways involved in low-temperature responses such as glycolysis, fatty acid metabolism, amino acid catabolism and oxidative phosphorylation were analyzed using PathVisio [41, 42] and Cytoscape [43] software. Genes up-regulated 2 fold or more were defined as red; genes down-regulated by 0.5 fold or more were defined as green.

### Total RNA extraction and real-time PCR

RNAs were extracted from brains, gills, livers, and kidneys of experimental fish (see description above) using a TriPure Isolation Reagent kit (Roche, Basel, Switzerland) To eliminate genomic DNA contamination, RNA pellets were dissolved in 30 μL of sterilized distilled and deionized water and treated with the RNA clean-up protocol from the RNAspin Mini RNA isolation kit (GE Health Care) following the manufacturer's instructions. Extracted RNA samples were stored at -80°C after isolation. The concentration and purity of extracted total RNAs were measured using a NanoDrop 2000 (Thermo). The integrity of total RNA was checked by agarose gel electrophoresis. Purified RNA with an A_260_/A_280_ ratio between 1.8 and 2.0 was used for real-time PCR analysis. First-strand cDNA was synthesized from 2 μg of total RNA using an iScript cDNA synthesis kit (Bio-Rad, Hercules, CA, USA) following the manufacturer's instructions. Expression levels of succinate dehydrogenase subunit A (SDHA) were quantified by MiniOpticon real-time PCR system (Bio-Rad Laboratories). Primers for real-time PCR to analyze SDHA expression were designed using Perlprimer software [44] and had the following sequences (5′ to 3′): forward GGAGGGATGTGATAAGATGG, and reverse CATACAATACCTCTGTCAAAGG; Elongation factor 1α (EF-1α), forward, CCATTGTTCAGATGATTCCCG and reverse, CTTCTTGATGACACCAACAGC. A standard curve was constructed for each target gene to optimize the template cDNA concentration and to verify that the threshold cycle (Ct) fell into an acceptable range. The amplification efficiency for standard curves of the primer pairs used for real-time PCR analysis was within the range 95–100%. The reaction mix for real-time PCR contained 8 μL of cDNA (20X dilution), 0.5 μM of primer pair, and 10 μL of 2X SYBR Green Supermix (Bio-Rad). The conditions for all real-time PCR amplifications were as follows: 95°C for 5 min, followed by 40 cycles of 95°C for 10 sec and 61°C for 20 sec. All samples were run in triplicate. Non-template control reactions were carried out using sterile deionized water instead of cDNA template to determine the background signal levels. A melting curve analysis was performed after each reaction to confirm the efficiency of the conditions for real-time PCR and to verify that no primer dimers or other non-specific products were synthesized during the reactions. EF-1α was used as an internal control for normalization of SDHA. For each unknown sample, the comparative Ct method was used to obtain the corresponding values of target gene where Ct corresponded to the threshold cycle number; this was calculated using the formula, 2^ [(Ct target, n—Ct EF-1α, n) —(Ct target, c—Ct EF-1α, c)] [45]

### Statistical analysis

In the 18°C acclimation experiments for SDHA expression, statistical significance was determined using student’s t-test (P<0.05) for group data analysis. Values are expressed as means ± S.E.M.

## Results

### Paired-end sequencing and *de novo* assembly

**Fig 1:**
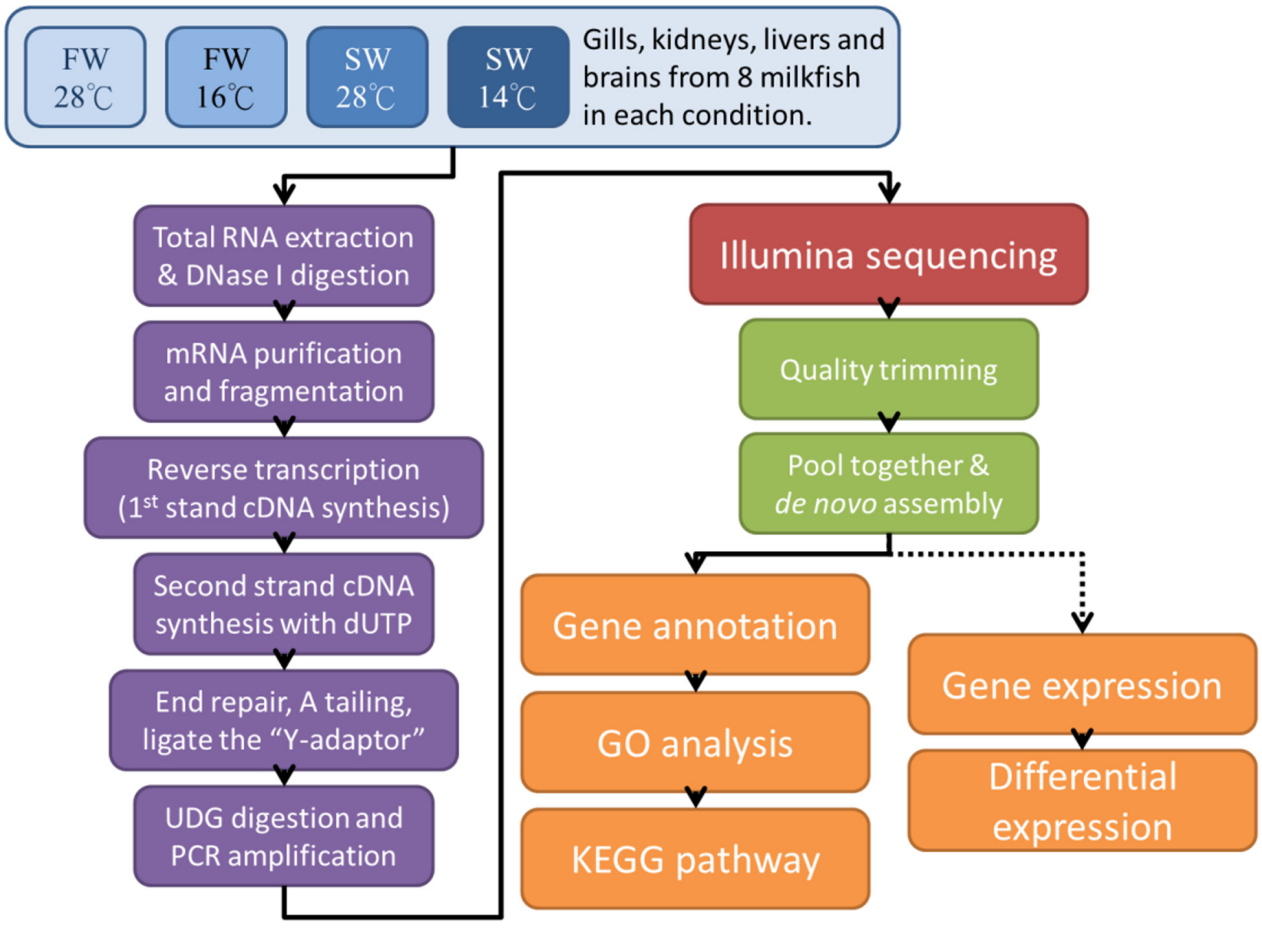
Fish were divided into four groups: freshwater at 28°C (FW28°C), seawater at 28°C (SW28°C), freshwater at 18°C (FW18°C) and seawater at 18°C (SW18°C). The red frames indicate differential expression comparisons between different temperatures while green ones represent the salinity effects.

**Fig 2:**
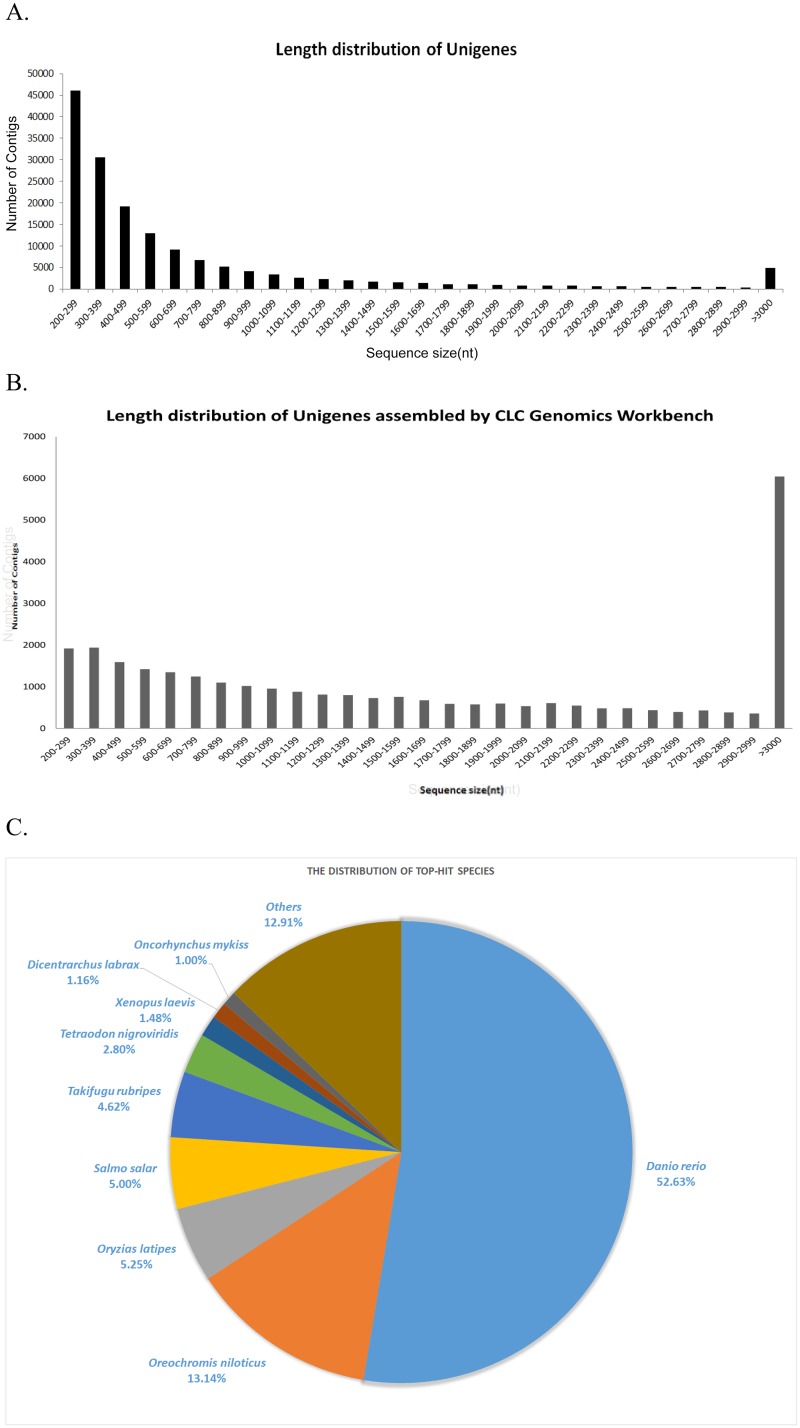
Length distribution of unigenes and the distribution of top-hit species. (A) Unigenes assembled from Trinity and Oases, (B) unigenes refined from CLC Genomics Workbench, and (C) the distribution of top-hit species.

To characterize the transcriptome of milkfish, total RNA samples isolated from the brain, gill, liver, and kidney subjected to library construction and high-throughput sequencing. The main steps for the data analysis were shown in Fig 1. Ten GB clean bases were generated for each condition. After trimming and filtering the raw reads, *de novo* assembly was performed. The *de novo* statistics of all contig sequences indicated the results from four conditions (i.e., FW/28°C, SW/28°C, FW/18°C and SW/18°C) by Trinity. To combine the four conditions, Oases was implied. The last application of CLC Genomics Workbench platform (CLCbio) plays a role in the assembly of complete coding sequence as much as possible. The performance of these tools was assessed and it was shown in Table 1 and S1 Table. The length distribution of assembled contig form Trinity and Oases were shown in Fig 2A, and refined sequences by CLC Genomics Workbench (Fig 2B).

**Table 1:** *De novo* assembly results.

	Contig sequences	Unigenes	Transcripts
Max contig length	27,742	11,893	28,119
Min contig length	200	200	200
Avg contig length	1,562.53	723.25	1,936
N50	2,584	1,057	3,023
Number of contigs	991,283	163,017	29,669
GC content	46.8%	44.2%	45.4%

In the blast against the Nr database, the E-value distribution of the top hits revealed that 26.28% of the matched sequences had a significant similarity with the majority of the E-values were distributed in the range of 1.0 × 10^−10^ to 1.0 × 10^−100^. The species distribution revealed that 52.63% of the hits matched sequences from *Denio rerio*, followed by *Oreochromis niloticus* (13.14%), *Oryzias latipes* (5.25%), *Salmo salar* (5.0%), *Takifugu rubripes* (4.62%), *Tetraodon nigroviridis* (2.80%), *Xenopus laevis* (1.48%), *Dicentrarchus labrax* (1.16%), *Oncorhynchus mykiss* (1.0%) and other species (12.91%) (Fig 2C).

### Functional annotation

**Fig 3:**
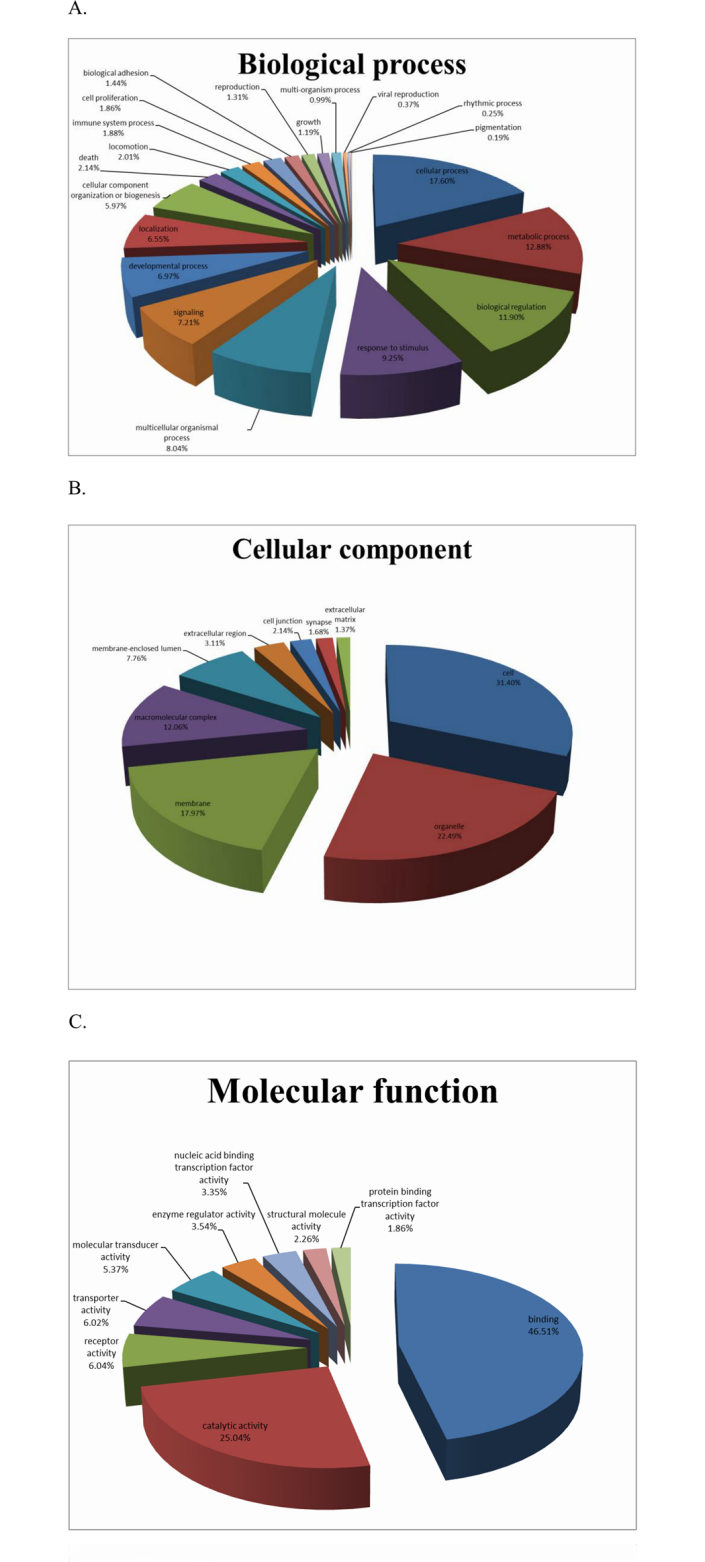
Classification of the annotated amino-acid sequences. Amino-acid sequences were grouped into different functional subcategories: (A) biological process (B) cellular component and (C) molecular function.

Gene ontology (GO) is an international standardized gene functional classification system which can be used to assign properties to genes and their products in any organism. Based on the results of Nr annotation, the GO annotations of unigenes were generated using the BLAST2GO program, and 29669 were annotated and assigned into three categories. The mapping rates of biological process, cellular component and molecular function was 86.33%, 46.67% and 28.38%, respectively. Among those assigned to the category of biological process, ‘cellular process’ (17.6%), ‘metabolic process’ (12.88%), and ‘biological regulation’ (11.9%) were most highly represented. The other unigenes were assigned into 18 other biological processes, including response to stimulus (9.25%), multicellular organismal process (8.04%), signaling (7.21%), developmental process (6.55%), localization (6.75%), cellular component organization or biogenesis (5.97%), death (2.14%). In addition, nine GO functional groups were assigned in the category of cellular component, with cell (31.4%) and organelle (22.49%) being the most highly represented. Similarly, nine GO functional groups were assigned in the category of molecular function, with binding (46.51%) and catalytic activity (25.04%) being the most highly represented (Fig 3).

**Fig 4:**
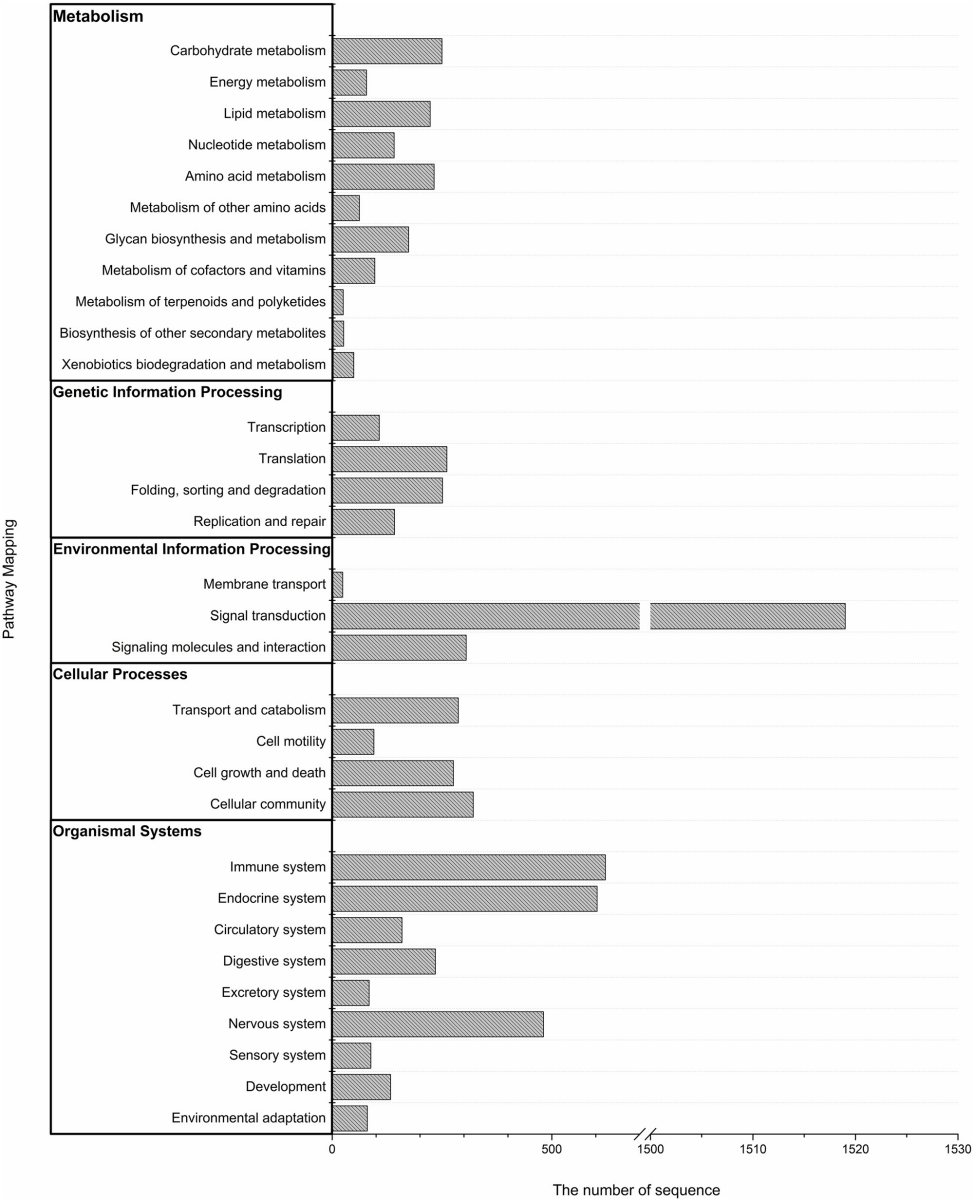
Distributions of the KEGG pathways. Putative proteins were mapped to the reference canonical pathways in the KEGG database. The bar chart shows the number of sequences in different pathway categories.

We also made use of the KEGG pathway database, which recorded networks of molecular interactions in cells, to help us learn more about the biological functions of the genes. To systematically analyze metabolic pathways and complicated biological behaviors, we classified the unigenes into biological pathways by mapping the annotated CDS sequences to the reference canonical pathways in the KEGG database. The mapping rate of KEGG was 31.27%. The 29669 unigenes were assigned to KEGG pathways: 608 were assigned to metabolic pathways; and 159 were assigned to biosynthesis of secondary metabolites, which are highly represented in the category of metabolism (Fig 4).

### Differential expression in the transcriptome after hypothermal and salinity stress, and KEGG pathway analysis

The FW- and SW-acclimated milkfish displayed different adaptation and compensation strategies in response to hypothermal stress. Cold-exposure-induced changes in mRNA abundance associated with carbohydrate metabolic pathways were found in presumptive glucose, fatty acid, and amino acid involvement in the TCA cycle and oxidative phosphorylation in the FPKM and KEGG analyses. The main changes in mRNA abundance in milkfish exposed to low temperature compared normal temperatures, in SW and FW, are given below:

#### Glycogen synthesis

**Fig 5:**
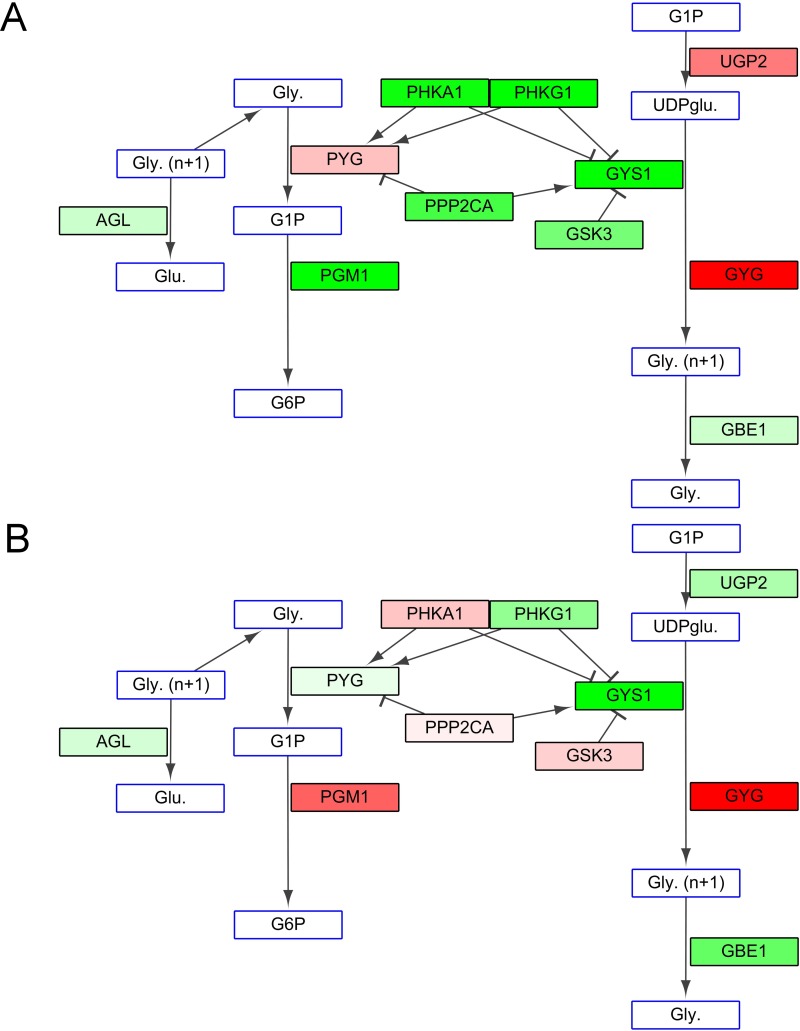
(A) Glycogen synthesis in fish in FW indicates down-regulation of glycogen synthase 1 (GYS1) and its regulator. (B) Glycogen synthesis in fish in SW also indicates down-regulation of glycogen synthase 1 (GYS1) and its regulator and the up-regulation of phosphoglucomutase 1 (PGM1), which is responsible for glycogen degradation. Metabolites or compounds are represented in rectangles with blue frames. Rectangles with black frames are mRNA expression of enzymes that participate in glycogen synthesis. Gene up- and down-regulation are represented by the FPKM ratio of 18°C to 28°C. Green indicate a decrease in expression of more than 0.5 fold (down-regulation); red indicates an increase of over 2-fold (up-regulation). The less intense shades of green and red indicate changes of expression level between 0.5 and 2 fold.

The most important genes involved in the glycogen synthesis pathway are glycogen synthase 1 (GYS1) and its regulators, phosphorylase kinase α1 (PHKA1), serine/threonine-protein phosphatase 2A (PPP2CA) and glycogen synthase kinase 3 (GSK3). The lower temperature reduced glycogen synthesis in FW and SW. With respect to glycogen degradation, the FW 18°C/28°C group showed up-regulation of glycogen phosphorylase (PYG), which is responsible for glucose 1-phosphate production (Fig 5A); the SW 18°C/28°C group showed up-regulation of phosphoglucomutase 1 (PGM1), which is responsible for glucose 6-phosphate production (Fig 5B). Thus, the glycogen synthesis pathway was down-regulated and the breakdown of glycogen was up-regulated in milkfish exposed to a hypothermal environment (Fig 5).

#### Glycolysis and gluconeogenesis

**Fig 6:**
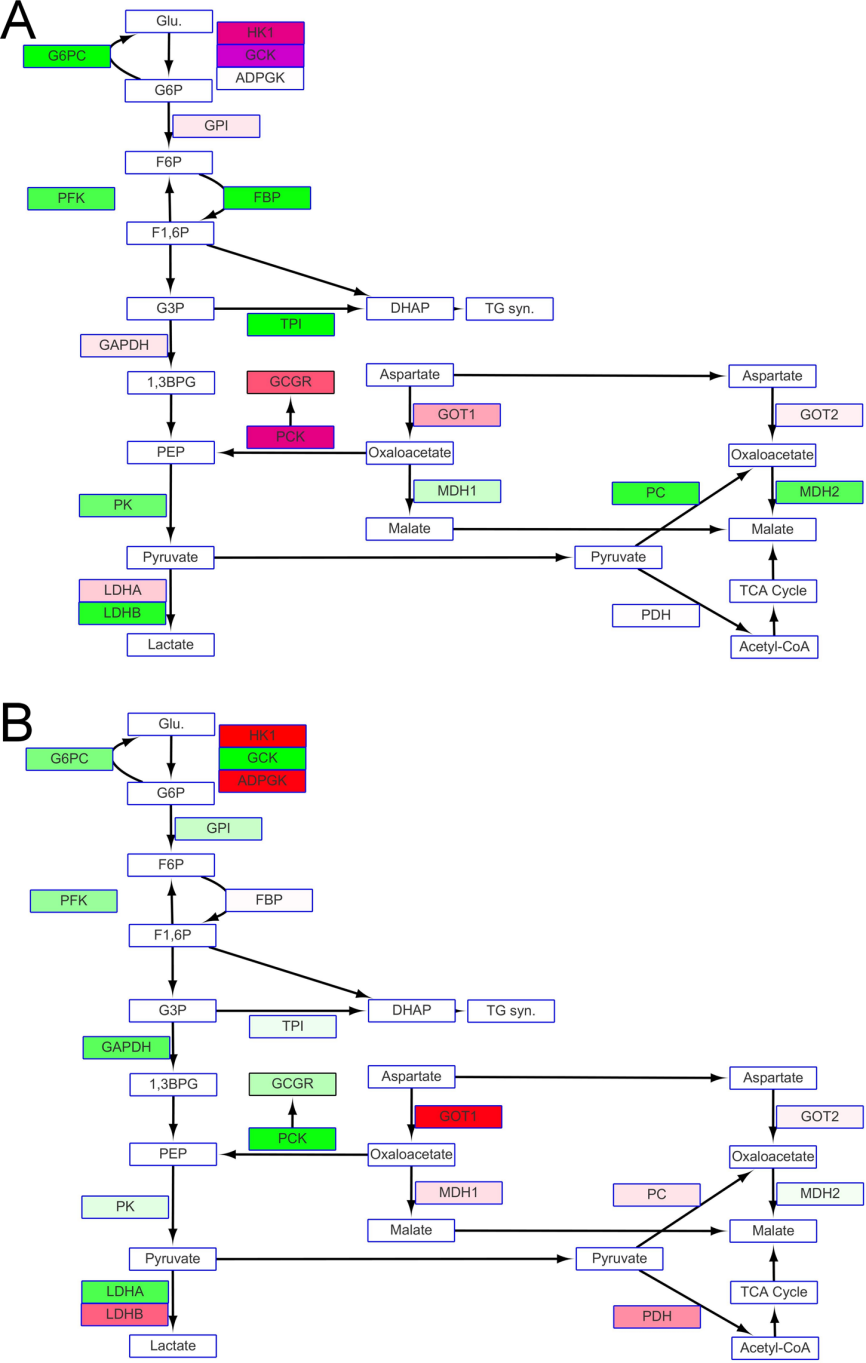
(A) Glycolysis in fish in FW at 18°C appeared vigorous because of the up-regulation of glucagon receptor, hexokinase (HK), glucokinase (GCK) and anaerobic LDHa, (B) Up-regulation of pyruvate carboxylase (PC), PDH and aerobic LDHb supported aerobic respiration in fish in SW at 18°C. Metabolites or compounds are indicated by rectangles with blue frames. Rectangles with black frames are mRNA expression of enzymes that participate in glycogen synthesis. Changes in levels of expression are shown as described in Fig 5; purple represents up-regulation of over 5 fold in the ratio of 18°C to 28°C.

The expression of genes encoding enzymes for glycolysis was up-regulated in both FW and SW milkfish under hypothermal stress. The magnitude of up-regulation differed, for example, glucokinase (GCK) was the highest in fish kept in FW but lowest in those in SW. More active glycolysis might be a consequence of the down-regulation of genes for gluconeogenesis, such as glucose-6-phosphatase and fructose 1,6-bisphosphatase. Differential expression of lactate dehydrogenase (LDH) isoforms was also found under cold stress: LDHA was up-regulated in FW fish, whereas LDHB was up-regulated in SW fish (Fig 6). A higher FPKM was observed for phosphoenolpyruvate carboxykinase (PCK) and glucagon receptor (GCGR) in FW milkfish exposed to hypothermal stress (Fig 6A). Up-regulation of pyruvate dehydrogenase (PDH), a key step for pyruvate entering the TCA cycle, was also found for SW fish (Fig 6B).

#### Fatty acid synthesis and degradation

**Fig 7:**
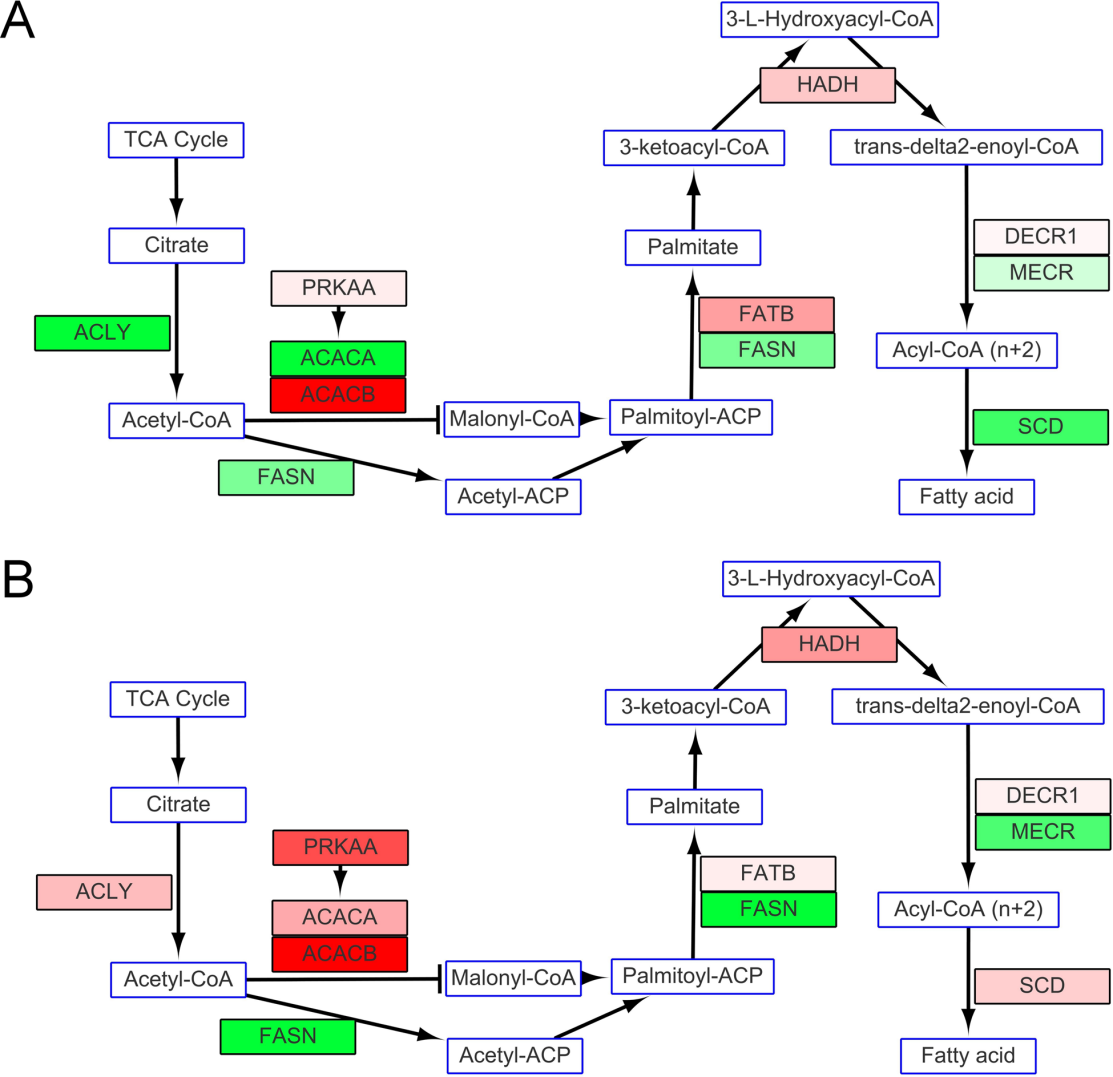
Fatty acid synthesis in fish in (A) FW and (B) SW Lower activities were present in fish under hypothermal stress because of the down-regulation of fatty acid synthase. Inhibition of fatty acid synthesis also presented as up-regulation of 5'-AMP-activated protein kinase α (PRKAA) and acetyl-CoA carboxylase 2 (ACACB). Metabolites or compounds are indicated in rectangles with blue frames. The rectangles with black frames show mRNA expression of enzymes that participate in glycogen synthesis. Changes in levels of expression are shown as described in Fig 5.

**Fig 8:**
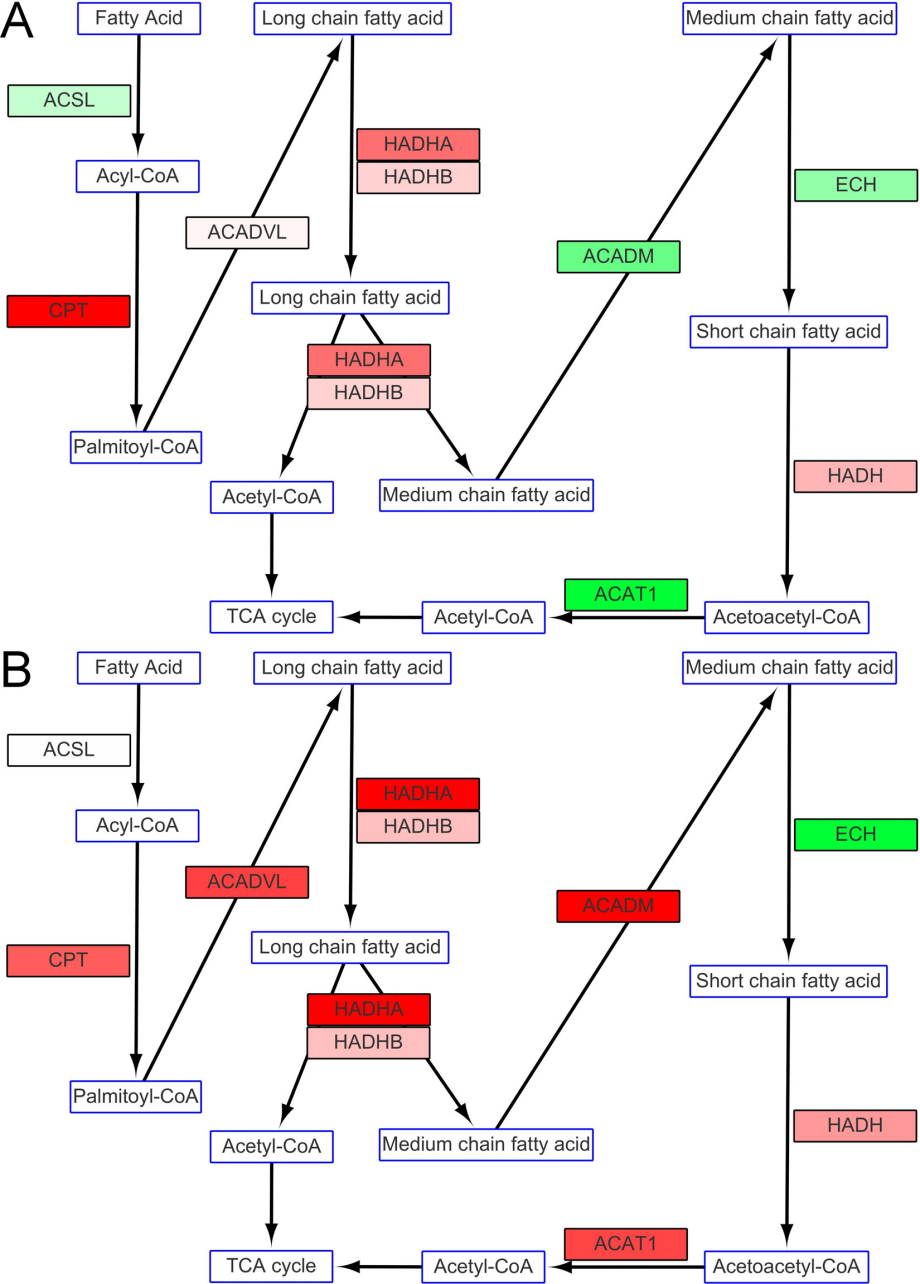
Fatty acid degradation in fish in (A) FW and (B) SW. Metabolites or compounds are indicated by rectangles with blue frames. Rectangles with black frames show mRNA expression of enzymes that participate in glycogen synthesis. Changes in levels of expression are shown as described in Fig 5.

Enzymes involved in fatty acid synthesis in cold-stressed milkfish were at similar levels to those of fish at the normal temperature except for the down-regulation of fatty acid synthase (FASN) expression (Fig 7). 5'-AMP-activated protein kinase α (PRKAA), acetyl-CoA carboxylase 1 (ACACA) and acetyl-CoA carboxylase 2 (ACACB), which are known inhibitors of fatty acid synthesis, were all up-regulated in fish in SW and under hypothermal stress (Fig 7B). However, all the enzymes that participate in fatty acid degradation were up-regulated in fish in SW at 18°C, while only long chain fatty acid degradation related genes were up-regulated in fish in FW at 18°C (Fig 8).

#### TCA cycle and amino acid catabolism

**Fig 9:**
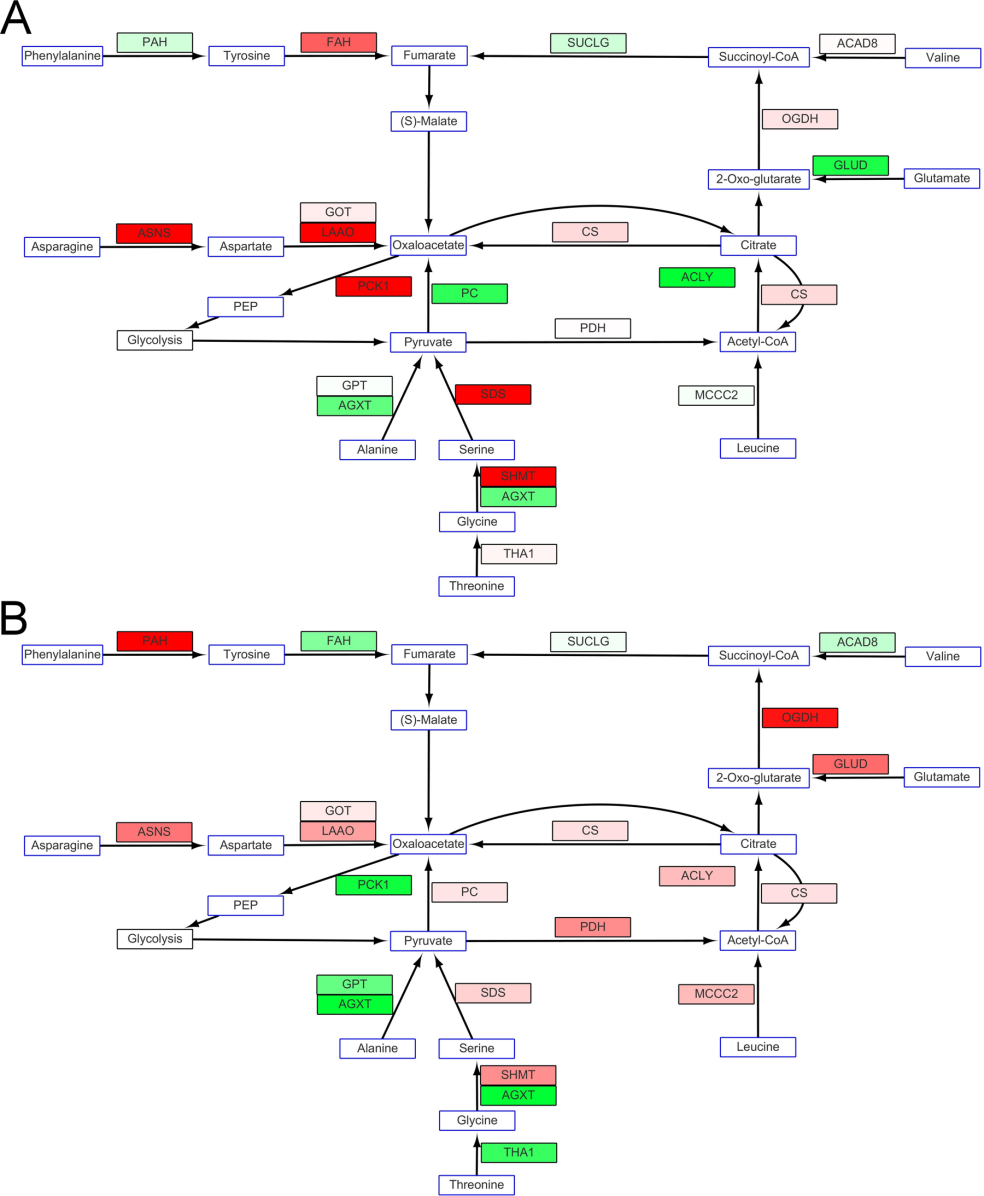
(A) The TCA cycle and amino acid catabolism in fish in FW were apparently altered for some specific amino acids, and the enzymes in TCA cycle were not up-regulated unless they were involved in the subsequent step of specific enzymes for amino acid catabolism. (B) In fish in SW at 18°C, mRNA expression of enzymes in the TCA cycle was mainly up-regulated. For NAD^+^ production, a higher ratio of up-regulation was found for mitochondrial nicotinamide nucleotide adenylyltransferase 3 (NMNAT-3) expression. Metabolites or compounds are indicated in blue frames. Black framed rectangles show mRNA expression of enzymes that participate in glycogen synthesis.Changes in levels of expression are shown as described in Fig 5.

The genes for enzymes involved in the TCA cycle showed slight up-regulation. At the same time, succinate dehydrogenase (SDH) involved in oxidative phosphorylation for the transport of electrons, was up-regulated to a greater magnitude in fish in SW than in those in FW. There are many metabolic pathways for amino acids to participate in the TCA cycle. Nine enzymes that incorporate particular amino acids into the TCA cycle were differentially expressed at the low temperature. Alanine transaminase (GPT) and alanine-glyoxylate transaminase (AGXT), which produce pyruvates that can be used for acetyl-CoA synthesis or gluconeogenesis, were down-regulated or not differentially expressed in fish in FW. However, L-serine/L-threonine ammonia-lyase (SDS), which transforms serine to pyruvate, was up-regulated. 3-methylcrotonyl-CoA carboxylase (MCCC2), which produces acetyl-CoA from leucine, was down-regulated in FW milkfish but up-regulated in those in SW. These opposing expression patterns may be evidence of differences in the mechanisms for low-temperature adaptation in SW and FW. Glutamate dehydrogenase (GLUD) uses 2-oxoglutarate, an intermediate of the TCA cycle, to catalyze nicotinamide adenine dinucleotide (phosphate) (NAD(P)H) production; expression of the enzyme were down-regulated in fish in FW. The TCA cycle and amino acid catabolism in fish in FW seemed to be affected by expression of the nine enzymes, and the enzymes in the TCA cycle were not up-regulated unless they were the subsequent step of specific enzymes for amino acid catabolism. By contrast, in SW milkfish, the levels of mRNA for enzymes in the TCA cycle were mostly up-regulated while the expression of genes for only five enzymes catabolizing four amino acids was up-regulated. For NAD^+^ production, a higher ratio of up-regulation was found for mitochondrial nicotinamide nucleotide adenylyltransferase 3 (NMNAT3) expression in fish in SW compared to those in FW (Fig 9).

#### Oxidative phosphorylation

**Fig 10:**
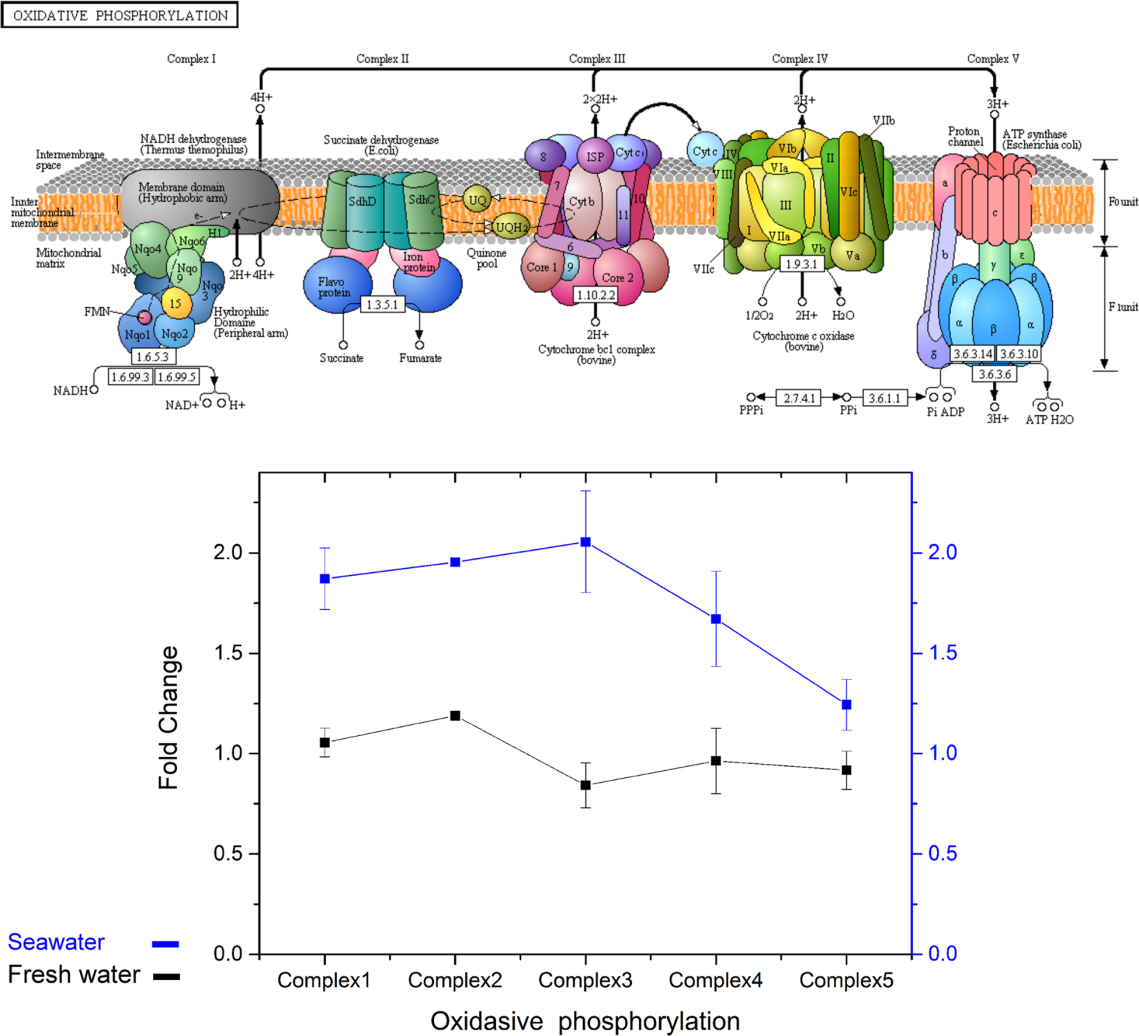
KEGG map of the oxidative phosphorylation pathway and the levels of complex expression. The error bars reflect differences in the expression of components of each complex. The black and blue lines represent the fold changes in expression of the ETC complex in fish in FW and SW, respectively, of the 18°C group compared to the 28°C group.

**Fig 11:**
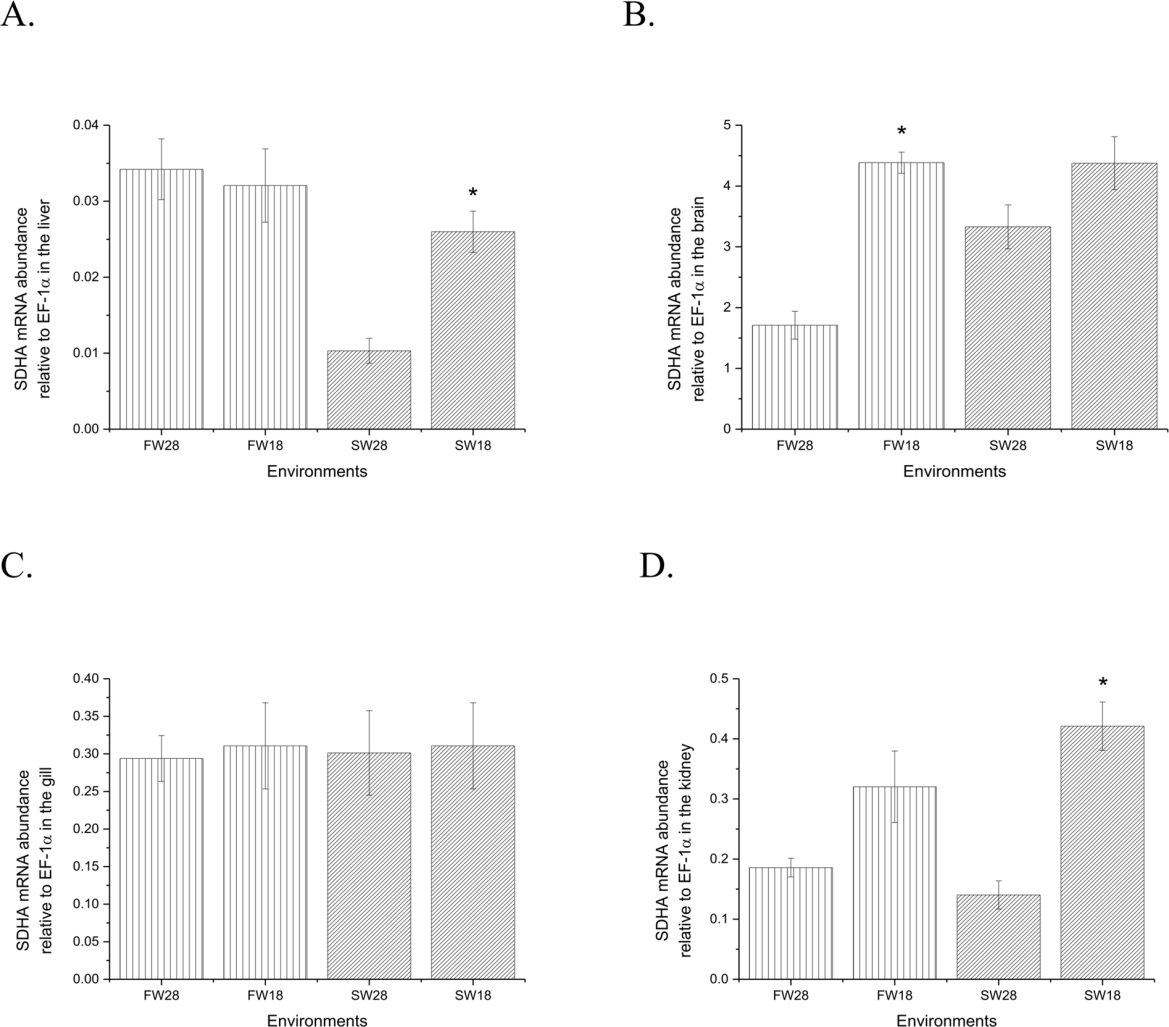
Relative abundance of succinate dehydrogenase subunit A mRNA in the liver (A), brain (B), gill (C) and kidney (D). N = 8 for each experiment. The asterisk indicates a significant difference by Student’s t-test (P < 0.05). Values are means ± S.E.M.

Oxidative phosphorylation represents the last step of energy production and the main source of ATP. Under hypothermal stress, the expression of all subunits in complex 1, 2, 3, and 4 were higher in the SW group; those in FW fish appeared to be unchanged (Fig 10). The error bars reflect differences in the expression of components in each complex, and are due to the very large subunits and unigenes in these complexes (Fig 10). In the flavin adenine dinucleotide (FAD) reduction steps, fumarate was produced by complex 2 in oxidative phosphorylation. In FW, fish at both temperatures showed similar patterns of complex 2 expression in the liver, gill, and kidney (Fig 11A, 11C and 11D) but up-regulation in the brain of the control group (Fig 11B). In fish in SW, SDHA was up-regulated in the brain and was significantly higher in the liver and kidney (Fig 10A, 10B, and 10D).

Overall, our results indicated a systemic up-regulation of gene expression related to basic energy metabolism in the SW group, whereas only genes for enzymes involved in glycolysis were up-regulated in the FW group along with those for specific amino acid catabolism.

## Discussion

RNA-seq has been increasingly applied to a wide spectrum of model and non-model animal species. The transcriptomes of several fish species such as lake sturgeon (*Acipenser fulvescens*) [46], European eel (*Anguilla anguilla*) [47], zebrafish (*Danio rerio*) [48], guppy (*Poecilia reticulata*) [49], silver carp (*Hypophthalmichthys molitrix*) [50], blunt snout bream (*Megalobrama amblycephala*) [51], common carp (*Cyprinus carpio*) [52], and Asian seabass (*Lates calcarifer*) [53], have been characterized by RNA-seq. The advantages of transcriptome sequencing are that it allows access at the genomic level when no genome information is available. The RNA can be pooled from many tissues and experimental treatments, thereby providing systemic access to the transcriptional characteristics of an organism. Through universal sequencing of the whole transcriptome, the transcript pool can also be normalized to screen for highly expressed genes, rare transcripts, and isoforms of particular genes [54]. Additionally, in the present study, the wide range of RNA-seq indicated that large amounts of sequences have been created for further investigation other than metabolism.

Milkfish is a marine species with excellent euryhalinity; therefore, the species is widespread and is commercially cultured using a wide range of salinities [55]. Milkfish are able to make energy modulations to achieve a more flexible and high metabolic rate in SW and brackish water [22]. Studies on salmon revealed that the activity of mitochondrial metabolic enzymes increase during smoltification in liver, gill, and kidney tissues [10–12]. During smoltification, a series of physiological changes occur to enable the juveniles to adapt from FW-living to SW-living. In the gills of smolts, two enzymes involved in glycolysis and six oxidative phosphorylation enzymes are up-regulated more than twofold [13]. For euryhaline fish in a SW environment, basal metabolism may be more sensitive and changeable than in fish in FW.

To date, various studies have found that cold adaptation commonly affects pathways for glucose metabolism and fatty acid degradation [25, 56, 57]. Thus, plasma glucose, lactate, and lipids are generally regarded as reliable parameters for measuring physiological responses in teleost fish experiencing hypothermia [20, 58]. In milkfish and grass carp, glucose levels increase rapidly upon exposure to cold treatment; they then decrease to the normal range within 2 days [25]. Changes in basic metabolism usually reflect switches in the energy supply source. Glucose, fatty acid and amino acids are well known energy providers. The switch from glycolysis or the TCA cycle to the oxidative phosphorylation pathway creates ATP as an energy source [59]. In the milkfish, changes in glucose and fatty acid metabolism have been reported following exposure to hypothermia [24, 25]. However, these studies did not investigate changes at the systemic or molecular levels. Enhancement of the limited capacity of stearoyl-CoA desaturase activity in milkfish suggested that the adaptation mechanisms of the fish in FW could not cope with hypothermal stress for a sustained period of time [24]. By comparison with the eurythermal grass carp, milkfish in FW alter their basal metabolic enzyme activities to participate in glycolysis/gluconeogenesis for a short period. The acute alterations in these metabolic enzyme activities [25] together with the FPKM changes found here provide strong evidence that milkfish under hypothermal stress tend to create more enzymes to compensate for their physiological homeostasis. The use of an NGS approach here provided further and more extensive data on basal metabolic gene expression under hypothermal stress.

Milkfish is a euryhaline teleost, which modified its physiology to adapt to environments of different salinities. However, osmoregulation is energy-consuming mechanisms and gill Na^+^, K^+^-ATPase activity decrease under low temperature [26]. Thus, systemic investigation on metabolic genes expression was provided to verify differences for low-temperature responses in seawater or freshwater milkfish. The speculations on metabolic pathways based on our KEGG analysis and the FPKM of NGS data allowed us to compare the transcriptomes of milkfish from the control and hypothermal groups. In the FW group, high expression of the genes for GCK, PCK, and L-amino-acid oxidase activity (LAAO) was found. GCK and PCK were known to be involved in glycolysis and gluconeogenesis [60], while LAAO produces oxaloacetate. Fumarylacetoacetase (FAH), which was up-regulated in the FW milkfish, might provide another source of fumarate for the TCA cycle. The increase in amino acid degradation for glycolysis may be an important mechanism for energy supply in the FW group. It is also possible that ATP created from glucose and amino acids in gluconeogenesis are principal energy providers in fish exposed to hypothermia [59]. However, the unchanged expression of oxidative phosphorylation complexes and the down-regulation of expression of acetyl-CoA providers, such as PDH, and acetyl-Coenzyme A acetyltransferase 1 (ACAT1), suggest that the amount of energy derived from the TCA cycle might be low in FW milkfish under hypothermia. In the SW group, hexokinase (HK), PDH and pyruvate carboxylase (PC) were up-regulated while PCK was down-regulated; thus, more acetyl-CoA and oxaloacetate might be produced in this group. The energy produced from the TCA cycle in the SW groups was apparently transferred to oxidative phosphorylation as we identified a slight up-regulation of PDH and ACAT1. Succinate in the TCA cycle provides electrons to complex 2 in oxidative phosphorylation for ATP synthesis. The highly up-regulated expression of alpha-ketoglutarate dehydrogenase (OGDH) and the systemic up-regulation of oxidative phosphorylation complexes 1–5 provided evidence that ATP synthesis and aerobic respiration were important strategies for the adaptation by SW milkfish to low temperature.

Up-regulation of oxidative phosphorylation complexes might also be due to the degradation of fatty acids. There is evidence that fatty acid degradation is likely induced in SW fish in hypothermal conditions but not in fish in FW, e.g., the increase in the activity of 3-hydroxyacyl-CoA dehydrogenase (HOAD) in the gilthead sea bream. This suggests that oxidation of free fatty acids contributes to ATP turnover at low temperatures [58]. The up-regulation of genes related to fatty acid degradation also provided reasonable evidence for the up-regulation of oxidative phosphorylation complexes. Our findings and speculations indicate that different strategies were utilized by SW and FW milkfish to compensate for energy loss in hypothermia. Systemic gene up-regulation was found in the SW group, but only genes related to glycolysis were up-regulated in the FW group. For gilthead sea bream maintained at a critically low temperature, the increase in LDH activity and the accumulation of lactate in tissues indicate that hypothermia stimulated the anaerobic component of the metabolism [58]. A similar phenomenon was also observed in FW milkfish under hypothermal stress. In zebrafish, SDH activity is regarded as aerobic capacity during thermal acclimation [5]. The expression of SDHA here revealed differential expression at 18°C between FW and SW milkfish. Since the gill is the major osmoregulatory organ, the unchanged expression of SDHA found in this study, together with the slightly elevated level in the gill of milkfish at 18°C [26], implied that hypothermia affected energy metabolism in milkfish but not osmotic homeostasis. In fish, one of the most important survival strategies for cold acclimation is an increase in the concentration of aerobic metabolic enzymes, such as the enzymes involved in the Krebs cycle and oxidative phosphorylation, which reside within the mitochondria [61]. Cold acclimation promotes changes in mitochondrial bioenergetics through increases in the levels of the enzymes related to energy synthesis, such as SDH, rather than increases in oxidative capacity [62]. In oxidative phosphorylation, succinate produced from TCA cycle represented the succession of glycolytic and degraded amino acid products. The up-regulated SDHA expression in glycolytic and amino acid pathway explains systemic enforcement of energy metabolism in the SW milkfish compared to FW milkfish. In addition, from the pathway speculation, 5 of 10 genes involved in fatty acid metabolism were up-regulated in FW, and all genes except enoyl-CoA hydratase (ECH) were up-regulated in SW under low temperature (Fig 8). Accordingly, we assumed that acetyl-CoA from fatty acid degradation might enter TCA cycle. Thus, fatty acid may also be one crucial energy supply in SW milkfish compared to that in FW individuals.

In summary, this study is the first to examine energy production by oxidative phosphorylation in milkfish and to show that this differed between fish in SW and FW under hypothermal stress. Milkfish may obtain more energy at lower environmental temperatures in SW than in FW. This is evidence that milkfish can adapt to lower temperatures in SW. Our analyses provided transcriptome sequences for a non-model fish, milkfish, as well as examples of different strategies for cold adaptation in FW and SW of a euryhaline fish species.

## Supporting Information

S1 Table Sequencing summary. (XLSX)

S2 Table Abbbreviations. (XLSX)

S3 Table Blastn to vertebrate. (XLSX)

S4 Table Gene ontology. (XLSX)

S5 Table Gene expressions in pathway speculation. (XLSX)
